# The Extracellular and Cytoplasmic Domains of Syndecan Cooperate Postsynaptically to Promote Synapse Growth at the *Drosophila* Neuromuscular Junction

**DOI:** 10.1371/journal.pone.0151621

**Published:** 2016-03-17

**Authors:** Margaret U. Nguyen, Jereen Kwong, Julia Chang, Victoria G. Gillet, Rachel M. Lee, Karl Gregory Johnson

**Affiliations:** 1 Molecular Biology Program, Pomona College, Claremont, California, United States of America; 2 Neuroscience Department, Pomona College, Claremont, California, United States of America; EPFL, SWITZERLAND

## Abstract

The heparan sulfate proteoglycan (HSPG) Syndecan (Sdc) is a crucial regulator of synapse development and growth in both vertebrates and invertebrates. In *Drosophila*, Sdc binds via its extracellular heparan sulfate (HS) sidechains to the receptor protein tyrosine phosphatase LAR to promote the morphological growth of the neuromuscular junction (NMJ). To date, however, little else is known about the molecular mechanisms by which Sdc functions to promote synapse growth. Here we show that all detectable Sdc found at the NMJ is provided by the muscle, strongly suggesting a post-synaptic role for Sdc. We also show that both the cytoplasmic and extracellular domains of Sdc are required to promote synapse growth or to rescue Sdc loss of function. We report the results of a yeast two-hybrid screen using the cytoplasmic domains of Sdc as bait, and identify several novel candidate binding partners for the cytoplasmic domains of Sdc. Together, these studies provide new insight into the mechanism of Sdc function at the NMJ, and provide enticing future directions for further exploring how Sdc promotes synapse growth.

## Introduction

During synapse development, an incoming neuronal growth cone transforms from a highly motile chemosensory structure into a pre-synaptic terminus, while the post-synaptic cell assembles the machinery required for the detection of neurotransmitter. At the neuromuscular junction (NMJ) synaptogenesis requires both forward and retrograde signaling between the presynaptic neuron and the postsynaptic muscle, and results in the formation of an exquisitely sensitive structure that translates incoming information in the form of action potentials into the contraction of muscle tissue. Following the initial phases of synapse formation, the NMJ exhibits a dramatic expansion in size concomitant with the expansion in volume of the muscle. Over the past two decades, the *Drosophila* NMJ has emerged as an outstanding model system in which to examine the molecular mechanisms both of synapse development and synapse growth (reviewed in [1]).

Heparan sulfate proteoglycans (HSPGs) are crucial regulators of synapse formation both in vertebrates and invertebrates (reviewed in [2, 3]). At the vertebrate NMJ, the HSPG Agrin is secreted by the presynaptic cell and induces the clustering of postsynaptic proteins, including acetylcholine receptors [4]. Agrin is not present in the *Drosophila* genome, but recent studies have shown that several other HSPGs act as regulators of NMJ formation [5, 6]. In particular, the transmembrane HSPG Sdc binds with nanomolar affinity to the receptor protein tyrosine phosphatase LAR to promote synapse growth [5]. Sdc mutants have a significant reduction in the number of boutons per synapse, whereas overexpression of Sdc increases synapse size [5]. Together, these data suggest that Sdc and LAR cooperate to promote synapse growth through direct interactions of their extracellular domains. With the exception of its interaction with LAR, however, the molecular mechanisms by which *Drosophila* Sdc promotes synapse growth have yet to be elucidated.

Further support for a conserved role for Sdc in regulating synapse development comes from vertebrates, where Sdc promotes the formation of dendritic spines through interactions with the cytoplasmic protein Synbindin [7, 8] and through phosphorylation by the receptor tyrosine kinase EphB2 [9]. Several other cytoplasmic binding partners have been identified for vertebrate Sdcs (reviewed in [10]), including ezrin–radixin–moesin (ERM) proteins, cortactin and Src [11] which bind to the membrane proximal conserved domain (C1); and syntenin, synectin, and CASK (reviewed in [12]) which bind to the membrane distal conserved domain (C2). These studies highlight an important functional role for Sdc’s cytoplasmic domain, and although similar binding partners might be hypothesized in *Drosophila*, due to the high degree of sequence conservation, none are yet known. In fact, recent studies have shown that the cytoplasmic domain of Sdc is entirely dispensable for Sdc function during axon guidance at the CNS midline [13] and dorsal branch cell migration [14].

In addition, the site of Sdc’s function at the *Drosophila* NMJ is not yet clear. Either presynaptic or postsynaptic expression of Sdc can enhance synapse size in *Drosophila* [5], and vertebrate HSPGs play crucial roles during synapse development on both presynaptic [15, 16] and postsynaptic sites [7–9, 17]. Therefore, in the present study we aim to explore the molecular mechanisms of Sdc function at the *Drosophila* NMJ. Using RNAi and overexpression techniques, we show that Sdc is localized to the NMJ exclusively through post-synaptic expression. We also demonstrate that both the cytoplasmic domains as well as the extracellular domains are required for Sdc’s synapse promoting activities. In addition, we identify by yeast two-hybrid screen, the first binding partners for *Drosophila* Sdc’s cytoplasmic domains. Together, these data provide insight into how Sdc functions to promote synapse growth at the *Drosophila* NMJ.

## Materials and Methods

### Genetics

*Drosophila* strains that were not viable to adulthood as homozygotes were balanced over CyO-[actin-GFP] or Tm6B-[ubi-GFP] as appropriate. Muscle- or neural-specific transgene expression was achieved with the GAL4-UAS system using 24B-GAL4 or elav-GAL4 (respectively) to express UAS-Sdc transgenes. Muscle- or neural-specific expression of Sdc-RNAi was achieved using the same GAL4 drivers, but using Sdc RNAi (VDRC ID#13322) from the Vienna *Drosophila* Resource Center. The wildtype UAS-Sdc construct (Sdc-wt) used in this study was used previously [5, 18, 19]. Sdc loss of function rescue experiments were performed as described previously [5] using *P2377/Df48*, *ubiSara* to provide the Sdc loss of function background. For the gain of function and rescue experiments, flies were raised at 20°C to reduce the penetrance of the lethality of muscle Sdc overexpression in the absence of endogenous Sdc.

### Immunohistochemistry

Wandering third instar larvae from sparsely populated bottles were collected and dissected in Ca2+-free saline. A detergent-free protocol was used to visualize labeling at the NMJ for anti-Sdc (1:250) [20], but all other antibodies including anti-HRP [1:2000] (Jackson Immunoresearch), anti-FasII [1:20] (Developmental Studies Hybridoma Bank), and anti-myc [1:3000] (Developmental Studies Hybridoma Bank) were used in the presence of 0.1% Triton as previously described [21]. Alexa488 goat anti-mouse, Alexa488 goat anti-rabbit, Alexa568 goat anti-rabbit and Alexa568 goat anti-mouse secondary antibodies (Invitrogen) were used at 1:500. Imaging was done on a Nikon C1 laser scanning confocal microscope. Statistical analysis of boutons per NMJ was conducted in Excel by Student’s t-test on larval pelts that were scored blind to genotype.

### Syndecan Construct Generation

Standard molecular cloning techniques were used to build the following Sdc constructs: Sdc-ΔCyto, Sdc-ΔEcto, Sdc-ΔCl, Sdc-ΔC2, Sdc-TM Swap, and Sdc-FL. Sdc-FL consists of the entire *sdc* gene. Sdc-ΔCyto, Sdc-ΔEcto, Sdc-ΔCl, and Sdc-ΔC2 lack the cytoplasmic domain, ectodomain, Cl domain, and C2 domain respectively. Sdc-TmSwap has the transmembrane domain of human platelet derived growth factor that lacks the dimerization abilities of Sdc’s own transmembrane domain [22]. Each construct was tagged with a 5xmyc tag that could be detected with immunostaining. All primers used for construct generation are shown in S1 Table.

The following steps outlined the general methodology for building constructs. First, the desired Sdc construct was amplified from a full-length Sdc template plasmid using PCR. The PCR product was then isolated by 0.8% agarose gel electrophoresis, then extracted and purified. To insert the Sdc construct into the final cloning vector, pUASTattB, it was excised using restriction enzymes, isolated via gel electrophoresis and gel extraction, and ligated into pUASTattB using T4 ligase. The ligated plasmid was transformed into DH5α competent cells and the plasmid was sequenced at Rancho Santa Ana Botanic Gardens. ~50μg of the construct in pUASTattB was sent to Genetic Services for transformation into the attP2 site of y, w flies on chromosome 3 (at position 68A4) using the ΦC31-based integrase system.

#### FL-Sdc

The FL-Sdc gene was amplified from Sdc-pUAST plasmid using PCR and the primers Not1 5’Sdc and Kpn1 3’Sdc. The PCR product was inserted into pCR^®^4-TOPO vector for cloning. The 5xmyc tag was extracted from pCS2-6MT vector via PCR. The PCR product was then inserted into the pCR4-TOPO vector. The 5xmyc tag was inserted into the XbaI site in the coding region of Sdc’s extracellular domain. The Sdc-5xmyc gene was then ligated into the pUASTattB vector.

#### SdcΔcyto and SdcΔC2

Primers (Not1 5’ Sdc and Kpn1 3’ dltCytoSdc) and (Not1 5’ Sdc and Kpn1 3’ dltC2 Sdc) were used to amplify the myc tagged SdcFL construct without the cytoplasmic or C2 domain to build the SdcΔcyto and SdcΔC2 constructs respectively.

#### SdcΔC1

Primers were used to obtain two PCR products from our SdcFL construct: (1) Not1-Xho1 (NX) segment, which contained the extracellular and transmembrane domains and (2) Xho1-Kpn1 (XK) segment, which contained the V and C2 domains (Table 1). The products were designed so that the first segment had Not1 and Xho1 restriction sites flanking its ends (5'->3') while the second segment had Xho1 and Kpn1 restriction sites at it ends (5'->3'). Each product was cloned into the pCR4-TOPO vector. The XK segment (made using XhoI 5' Sdc DT C1 and Kpn1 3' Sdc) was ligated into the pBS vector between Xho1 and Kpn1 cloning sites. The NX segment (made using Not1 5' Sdc and XhoI 3' Sdc DT C1) was also ligated into the same pBS vector between the Not1 and Xho1 sites to obtain the SdcΔCl construct. It was then extracted and inserted into the pUASTattB vector.

**Table 1:** Candidate binding partners from yeast two-hybrid screen.

Gene Identified^a^	n	Key Information
Cheerio (filamin)	6	Enriched in the CNS [26]
Eif4a*	4	Ubiquitous expression during embryogenesis [27]
Papilin	4	Extracellular protein [28]
CG9083	3	
EF2*	3	Translation elongation factor [29]
Glutamate Receptor Interacting Protein (GRIP)*	3	Expressed in muscles [30]
ELMO / Ced-12*	2	Ubiquitous during embryogenesis [31]
Coracle*	2	Expressed in muscle binds to Glutamate Receptor IIA [32]
CG10005	2	
CG7173	2	
Daughters against DPP (DAD)*	2	Involved in NMJ development [33]
Sallimus / Titin*	2	Involved in muscle development [34]
Sec13	2	Likely nuclear [35]
Twin of M4	2	Not in mesoderm [36]

^a^Genes identified in the yeast two hybrid screen and the number of times each gene was identified (n) are shown.

An asterisk indicates proteins that are known to be located in the muscle cytoplasm [25].

#### SdcΔecto

The primers (XhoI5' Tm&C SdtEC and Kpn1 3' Sdc) were used in PCR to amplify our SdcFL construct without its ectodomain and insert flanking Xho1 and Kpn1 restriction sites (5'->3') (Table 1). A set of primers (NotI 5' SMK SdtEC and XhoI3' SMK SdtEC) was used to amplify via PCR the ER signal signal sequence, 5xmyc gene, and Kozak sequence (SS+5xMyc+Kzk) from CS2+SS+5MT+Kozak plasmid. Both PCR products were cloned into the pCR4-TOPO vectors. The resulting plasmid was extracted and ligated into the pUASTattB vector.

#### Sdc-TM Swap

The transmembrane domain of human platelet derived growth factor receptor (hPDGFR) was built using oligonucleotides that corresponded to its sequence and the sequence of its complementary strand (3'PstIPdGFRXhoI and 5'PDGFRoligomer). Flanking Xho1 and Pst1 restriction sites were added. These oligonucleotides were annealed by adding equimolar amounts of the two oligonucleotides into Annealing Buffer (10mM MgCl2, 50mM NaCl, 20mM Tris) and subjecting the solution to the following conditions: (1) 2 min at 95°C, (2) 2 min at 72°C, (3) 2 min at 37°C and (4) 2 min at 25°C. The annealed product, which would be the double-stranded DNA of hPDGFR transmembrane domain with flanking restriction sites, was cloned and inserted into Sdc FL. The recombinant product was excised and ligated into pUASTattB.

### Yeast Two Hybrid Screen

The Matchmaker Two-Hybrid System 3 (Clontech) was used to conduct a yeast two-hybrid screen. To make constructs for use with this system, we designed primers to amplify cytoplasmic *Sdc* from existing plasmids with EcoRI/XhoI restriction sites at the N and C terminal ends (primer sequences Y2HSdcF: gcttatGAATTCtaccgcatgaggaaaaag, Y2HSdcRBait: ctcaccCTGCAGtcaggcgtagaactcgcg). The PCR product was ligated into pGEM-T (Promega) and sequenced. The pGEM-T with cytoplasmic *Sdc* was cut with EcoRI and XhoI and the *Sdc* fragment was purified (Micron Ultrafree-DA DNA Extraction From Agarose kit). This fragment was into the multiple cloning sites (MCSs) of pGBKT7 (pGBK). This plasmid was then amplified in DH5α bacteria and chemically transformed into AH109 yeast (genotype: MATa, trp1-901, leu2-3, 112, ura3-52, his3-200 gal4Δ, gal80Δ, LYS2::GAL1_UAS_-GAL1_TATA_-HIS3, GAL2_UAS_-GAL2_TATA_-ADE2, URA3::MEL1_UAS_-MEL1_TATA_-lacZ). The transformed yeast were plated on media lacking adenosine and histidine to ensure that the plasmids did not activate reporter genes.

A library containing cDNA fragments of *Drosophila* embryonic mRNA in pACT2 vectors (Clontech) was screened. The library was first amplified in a 5.5L bacterial culture from the library cells and plated onto 300 15mm LB+amp plates. The cells were allowed to grow until confluence at 30°C, and were then scraped into a total volume of 1.5L LB+amp. Plasmids were isolated with a Plasmid Mega Kit (Qiagen). The isolated pACT2 plasmids were chemically transformed into an AH109 yeast strain previously transformed with pGBKT7-cytoplasmic *Sdc*. Three yeast two-hybrid screens were conducted on 0.3mg of library plasmid, each with different aliquots of the amplified pACT2 plasmid library. For each screen, yeast were plated onto low stringency plates (–Leu –Trp) to calculate clones screened. The remaining cells were plated onto high stringency plates (–Leu –Trp –Ade –His). All media included ampicillin to prevent bacterial contamination. Plates were incubated at 30°Cfor 6–7 days, and colonies growing on high stringency plates were restreaked onto fresh high stringency plates to prepare for plasmid isolation.

The Clontech Yeast Plasmid Isolation Kit (YPIK) was used to isolate plasmids from yeast colonies growing on high stringency plates. PCR was performed on the resulting plasmids using primers designed to amplify the pACT2 insert (sequences 3’SeqRevpACT2 agtgaacagcggggtttttcagtatct, 5’SeqFwdpACT2 ctattcgatgatgaagataccccaccaa). The PCR products were analyzed on 0.8% agarose gels run at ~100V, and were sequenced at the Rancho Santa Ana Botanic Gardens. BLAST on Flybase was used to analyze the insert cDNA sequences (www.flybase.bio.indiana.edu/blast/). To ensure that these clones, inserted into the pACT2 library plasmid (which carries a Leu marker), did not independently activate reporter genes, we grid-plated the yeast two-hybrid transformants onto (–Leu) media with no selection pressure for the binding domain-Sdc vector (which carries a Trp marker). Replica plates of these grid plates onto (–Leu –Trp) and (–Leu –Ade –His) showed that many of the replica-plated colonies could not grow on (–Leu –Trp) plates, most likely because they had lost the binding domain-Sdc vector. None of the colonies that did not grow on (–Leu –Trp) grew on the (–Leu –Ade –His) plates.

## Results

**Fig 1:**
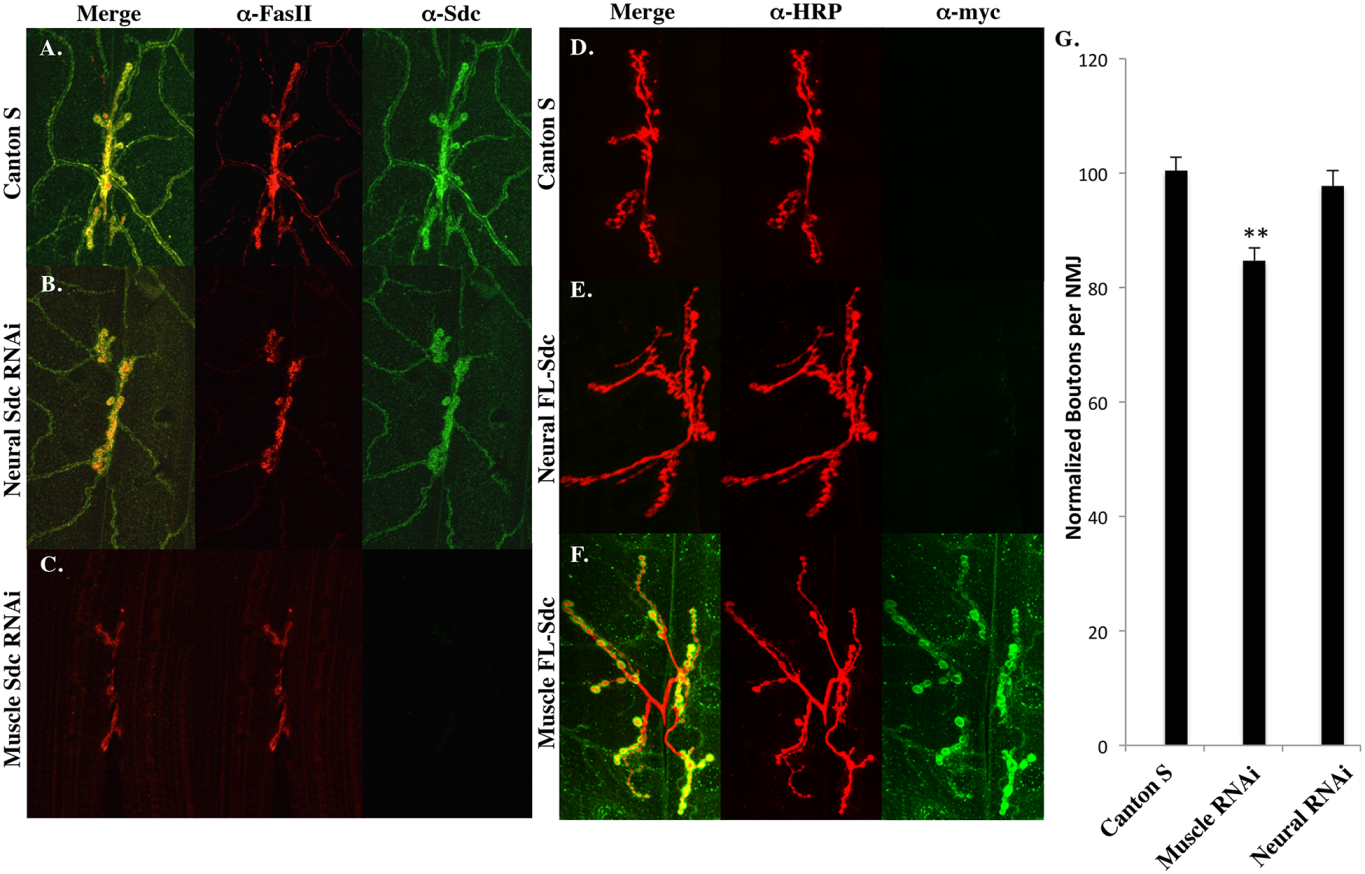
Sdc at the larval NMJ is provided postsynaptically. (A) In wildtype larvae, Sdc protein colocalizes with Fasciclin II at the 3^rd^ instar larval NMJ on muscles 6/7. (B) Neural expression of a Sdc RNAi construct using elav-Gal4 has no effect on synaptic Sdc, but muscle Sdc RNAi expression using 24B-Gal4 (C) completely abolishes the synaptic localization of Sdc. (D) Wildtype larvae have no detectable anti-Myc immunoreactivity. (E) When a myc-tagged UAS-SdcFL transgene is overexpressed in neurons using elav-Gal4, the protein does not localize to the larval NMJ. (F) When expressed in muscles using 24B-Gal4, this construct recapitulates the wildtype distribution of Sdc. (G) Postsynaptic, but not presynaptic, expression of a Sdc RNAi construct decreases synapse size when compared to Canton S controls (p < .01 n = 33; p > .05 n = 28). Bouton numbers for all graphs are normalized to Canton S (100).

Previous studies have shown that either pre-synaptic or post-synaptic expression of a full-length Sdc transgene can increase the size of the larval NMJ [5]. In order to determine whether the endogenous Sdc is localized to the synapse by expression in the muscle or in the neuron, we knocked down Sdc expression using tissue-specific Gal4 lines and a Sdc RNAi construct. In wildtype 3^rd^ instar larvae, an anti-Sdc antibody [20] shows Sdc protein localized to both the synapse and the tracheal/muscle interface (Fig 1A). Neural expression of a Sdc RNAi construct had no effect on Sdc localization (Fig 1B), but muscle expression of the same Sdc RNAi construct completely eliminated Sdc protein from both the synapse and the tracheal/muscle interface (Fig 1C).

Because Sdc mutants have decreased synapse size when compared to wildtype controls [5], we hypothesized that RNAi-mediated knock-down of Sdc would also result in decreased synaptic size. To test this hypothesis, we analyzed synapse size following either presynaptic or postsynaptic expression of the Sdc RNAi constructs. Postsynaptic expression of Sdc RNAi caused a significant 15% reduction in synapse size (p < .01 n = 34) whereas presynaptic expression of this same construct had no effect on synapse size (p > .05 n = 29; Fig 1G), indicating that postsynaptic Sdc is required for proper synapse growth.

These data suggest that the Sdc present at the NMJ is delivered by the muscle and not the neuron. To confirm this hypothesis, a myc-tagged full-length Sdc transgene (Sdc-FL) was overexpressed in either muscles or neurons, and the overexpressed protein was detected using an anti-myc antibody (9E10). Neural expression of Sdc-FL showed no accumulation at the synapse (Fig 1E) despite robust expression in the larval CNS and along motor axons (data not shown), but muscle expression of Sdc-FL recapitulated the normal distribution of Sdc on muscle 6/7 (Fig 1F). Together, these data show that the Sdc found at the larval NMJ is expressed post-synaptically.

**Fig 2:**
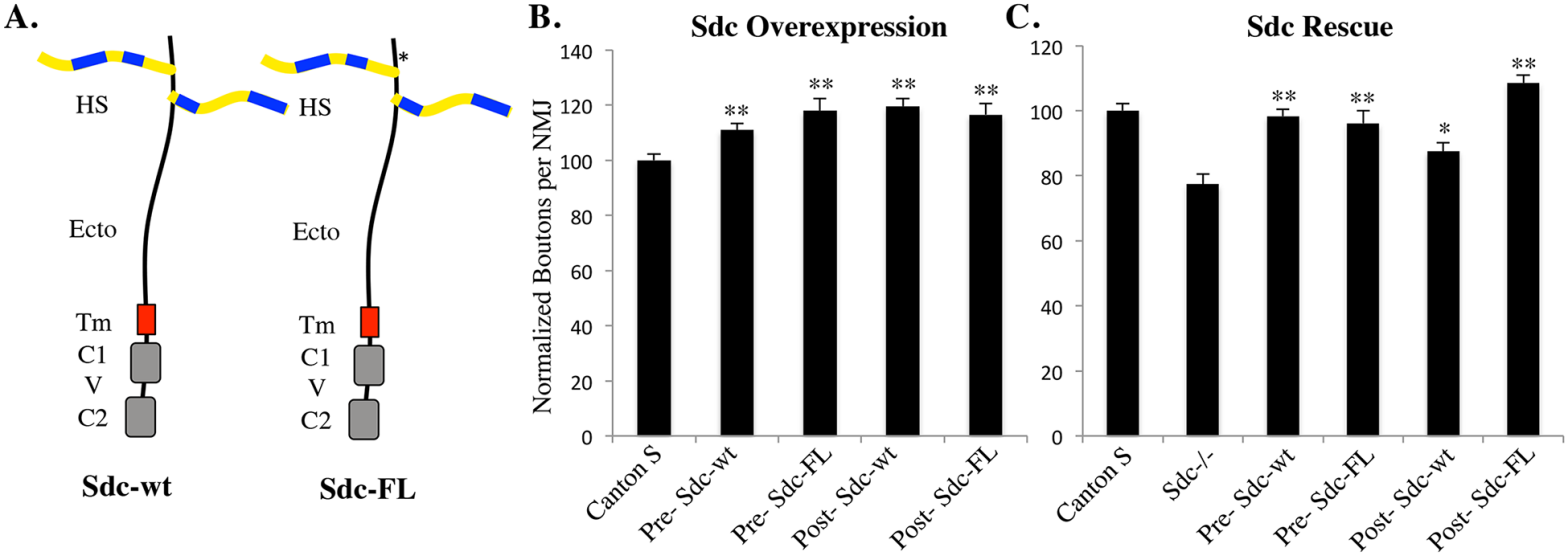
Sdc can promote NMJ growth when expressed in either neurons or muscles. (A) Schematic diagram of the wildtype Sdc construct (Sdc-wt) used in previous studies and the myc-tagged full-length Sdc construct (Sdc-FL) used in this study. HS—heparan sulfate sidechains; Ecto—extracellular domain; Tm—transmembrane domain; C1—Conserved Domain 1; V—Variable Domain; C2—Conserved Domain 2. *—site of 5xMyc tag used to detect construct expression. (B) Presynaptic or postsynaptic overexpression of either Sdc-wt or Sdc-FL causes a significant increase in the number of boutons at the NMJ on muscles 6/7, ranging from an 11% increase (presynaptic expression of Sdc-wt) to 19% (postsynaptic expression of Sdc-wt; **—p < .01) (C) Expression of either Sdc-wt or Sdc-FL in a Sdc mutant background significantly rescues the size of the 6/7 NMJ (**—p < .01, *—p < .05). These constructs are indistinguishable when expressed presynaptically, but Sdc-FL (cloned into the attp2 site) generates a more complete rescue than Sdc-wt when expressed postsynaptically. Sdc-wt expression data were published previously [5].

Previous studies indicate that either presynaptic or postsynaptic overexpression of a wildtype Syndecan construct can increase synapse size [5]. To confirm that the myc-tagged construct cloned into the Attp2 site (Sdc FL) acts similarly to the wildtype Sdc construct (Sdc-wt) used previously, we quantified synapse size following overexpression of Sdc either pre- or postsynaptically. Overexpression of Sdc-FL increases synapse size when expressed either pre- or post-synaptically (Fig 2B) similar to the Sdc-wt transgene [5]. Previous data shows that presynaptic expression of Sdc-wt rescues the Sdc mutant phenotype more completely than postsynaptic Sdc expression [5]. Using Sdc-FL construct cloned into the Attp2 site, both pre- and postsynaptic expression could completely rescue the Sdc mutant phenotype. These data suggest that Sdc can promote synapse growth when expressed either presynaptically or postsynaptically, and that the presence of the myc tag does not inhibit transgene function.

**Fig 3:**
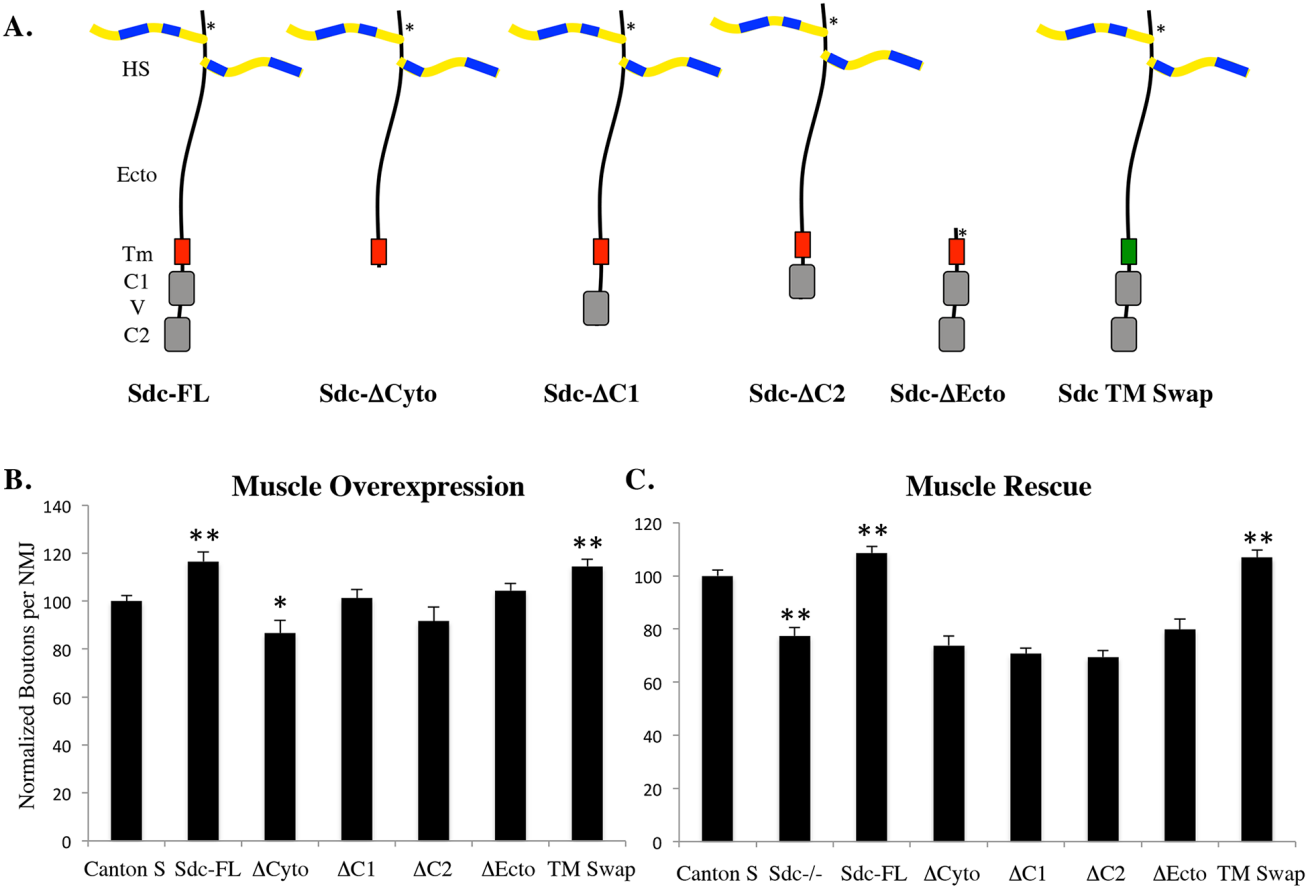
The cytoplasmic and extracellular domains are required for Sdc function. (A) Schematic diagram of the Sdc constructs used in this study. The Tm domain of the TM swap construct was made from human platelet derived growth factor receptor (hPDGFR). *—site of 5xMyc tag used for construct detection. (B) Muscle overexpression of the UAS-Sdc constructs shown in (A) using the 24B-Gal4 driver. Overexpression of either Sdc-FL or TM Swap generated a significant increase in the number of boutons per NMJ when compared to Canton S controls (p < .01 n = 21; p < .05 n = 21). Overexpression of ΔCyto caused a significant reduction in the number of boutons per NMJ (p < .05; n = 23). (C) Sdc mutants had a significant decrease in boutons per NMJ as described previously [5]. The UAS-Sdc constructs shown in (A) were expressed in muscles in a Sdc mutant background (P2377/Df48; ubi-Sara) using the 24B-Gal4 driver. Both Sdc-FL and TM Swap constructs generated a significant rescue of the number of boutons per NMJ when compared to Sdc mutants (p < .01 n = 40; p < .01 n = 23).

Having demonstrated that muscle tissue is the source of synaptic Sdc, we next set out to determine the domains of Sdc that are required for its function. *Drosophila* Sdc, like vertebrate Sdcs, consists of an extracellular domain with little sequence conservation outside of the heparan sulfate attachment sites [23] a highly conserved transmembrane domain that promotes dimerization [22], and two highly conserved cytoplasmic domains (C1 and C2) flanking a variable domain (V; reviewed in [10]). To probe which of these domains are required for Sdc function in vivo, we generated and expressed an array of myc-tagged Sdc transgenes lacking one or more of these domains (Fig 3A) in muscle tissue. Overexpression of Sdc-FL caused a significant 17% increase in synapse size on muscle 6/7 (p < .01; Fig 3B), consistent with previous literature [5]. Of the mutant constructs tested, only Sdc Tm-swap was capable of generating a significant increase in synapse size (p < .01) when overexpressed (Fig 3B). In fact, overexpression of a construct lacking the cytoplasmic domains resulted in a small but significant decrease in synapse size (p < .05).

In order to determine which domains of Sdc were necessary to rescue the Sdc mutant phenotype, we expressed these constructs in a Sdc mutant background. The loss of Sdc caused a significant 23% reduction in the size of the synapse (p < .01 n = 35), consistent with previous literature [5]. When expressed this background, Sdc-FL and Sdc-Tm swap were able to completely rescue the Sdc mutant phenotype (p < .01; Fig 3C), but none of the constructs lacking either the extracellular or cytoplasmic domains could. These data suggest that both the cytoplasmic and extracellular domains are required for Sdc’s ability to promote synapse growth, but that the dimerization potential of Sdc’s transmembrane domain appears to be dispensable in this context.

**Fig 4:**
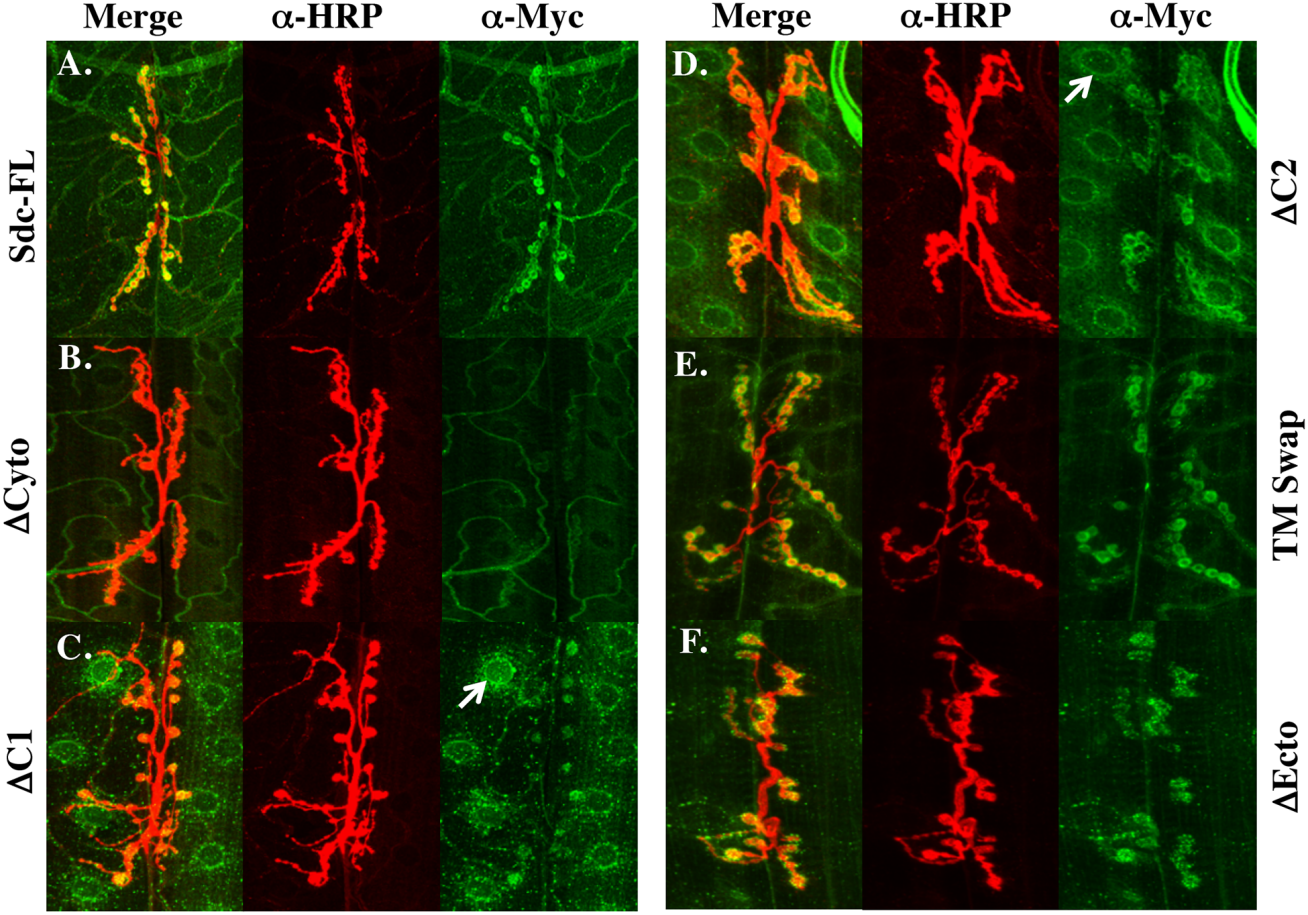
Sdc’s cytoplasmic domain influences Sdc’s subcellular localization. (A) In wildtype larvae, Sdc colocalizes with HRP at the 3^rd^ instar larval NMJ on muscles 6/7. (B) Muscle expression of a Sdc ΔCyto construct has diminished synaptic localization whereas expression of a construct lacking either C1 (C) or C2 (D) show intracellular accumulation of protein (arrows) in addition to the synaptic localization. Both the TM Swap construct (E) and the ΔEcto construct (F) exhibit similar subcellular localization to Sdc-FL.

The extracellular and cytoplasmic domains might be required for Sdc function because they play an instructive role in promoting synapse growth, or because they are simply required for localizing Sdc to the synapse. In order to determine how the extracellular and cytoplasmic domains of Sdc might influence Sdc function, we first examined the subcellular distribution of the Sdc constructs when expressed using a muscle driver. The overexpressed full length Sdc protein is concentrated around type 1 boutons at the 6/7 NMJ (Fig 4A) similar to wildtype Sdc (Fig 1A). A construct lacking the cytoplasmic domain has diminished synaptic localization (Fig 4B), whereas constructs lacking either C1 or C2 tend to accumulate in perinuclear structures (Arrows; Fig 4C and 4D), but also show substantial synaptic localization. The TM swap construct and the ΔEcto construct exhibited grossly normal synaptic distributions (Fig 4E and 4F). These data led us to hypothesize that the extracellular and transmembrane domains of Sdc are not required for synaptic Sdc localization, and that trafficking Sdc through the secretory pathway and localization to the synapse is driven largely by Sdc’s highly conserved C1 and C2 domains.

**Fig 5:**
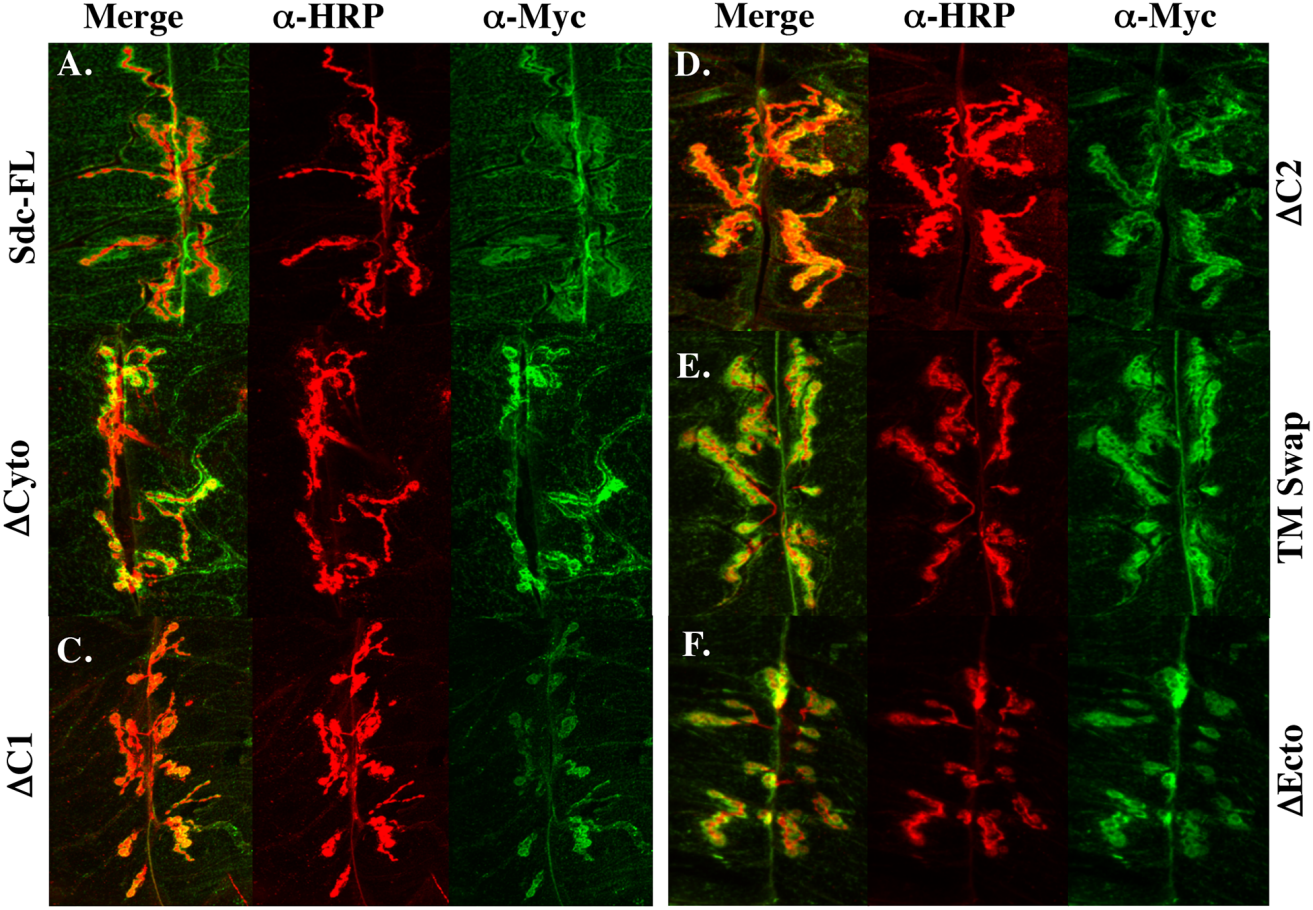
Detergent-free staining shows all Sdc constructs localize to the synapse. Using a detergent-free staining protocol to visualize only the cell-surface distribution of the expressed Sdc transgenes, all Sdc constructs appear to localize to the synapse (A-F) although there may be a slight decrease in staining intensity with the ΔC1 construct (C).

To examine this hypothesis further, we utilized a detergent-free staining protocol that allowed us to focus on the distribution of the mutant Sdc proteins exclusively at the cell surface without the risk of extracting detergent-soluble proteins. Although this staining protocol generated more variable results in terms of staining intensity, all constructs localized to the synapse under these staining conditions (Fig 5), including both ΔCyto and ΔEcto, although there appeared to be a trend toward reduced cell surface expression of Sdc with the ΔC1 construct (Fig 5C). Due to the high variability in staining intensity, this trend could not be quantified. Nevertheless, these data suggest that neither the extracellular domain nor the cytoplasmic domain of Sdc is required to localize Sdc to the synapse, and that once at the cell surface, synaptic localization of Sdc can be driven by either domain. Together, these data suggest that the cytoplasmic domains of Sdc might facilitate the passage of Sdc through the secretory pathway, but that once at the cell surface, the cytoplasmic domains are not required to bring Sdc to the synapse.

Synaptic binding partners for the extracellular domains of *Drosophila* Sdc have previously been identified [5, 24], but no cytoplasmic interactors are known for *Drosophila* Sdc. To identify binding partners for the cytoplasmic domains of *Drosophila* Sdc, we conducted a yeast two-hybrid screen on a *Drosophila* embryonic cDNA library using the cytoplasmic domains of Sdc as bait. Screening through 850,000 cDNA clones, we identified 580 cDNAs that encoded candidate Sdc binding partners. Of these, 134 were sequenced, and 80 of these sequences matched *Drosophila* genes (S2 Table). Fourteen *Drosophila* genes were obtained multiple independent times in this screen (Table 1) suggesting a potential interaction with the cytoplasmic domains of Sdc. Several of these candidate binding partners are expressed post-synaptically and suggest promising future directions for research.

## Discussion

In this study we demonstrate that *Drosophila* Sdc is localized to the NMJ through postsynaptic expression, and that both the cytoplasmic domains and the extracellular domains are required for Sdc’s ability to promote synapse growth. The potential for dimerization mediated by the transmembrane domain of Sdc, however, is not required for Sdc function. We also identify the first candidate binding partners for the cytoplasmic domains of Sdc in *Drosophila*. Together, these data suggest promising new directions to pursue Sdc function at the larval NMJ.

Previous studies demonstrated that either pre- or postsynaptic overexpression of Sdc can increase the size of the larval NMJ, and that presynaptic expression of a Sdc transgene can fully rescue the Sdc mutant phenotype [5]. This is a particularly surprising finding, given that no detectable Sdc reaches the NMJ when expressed with a neural driver (Fig 1E). Combined with the data in this study showing that postsynaptic expression of a Sdc-FL transgene generates a robust gain of function phenotype, and can fully rescue the Sdc mutant phenotype, this suggests that Sdc has distinct synapse growth promoting activities in the neuron and in the muscle. While we were able to confirm the ability for presynaptic Sdc expression to increase synapse size in this study, the mechanism by which Sdc acts presynaptically is not understood. It is possible that elevated expression of Sdc at the neuronal cell body increases the activity of the motor neurons innervating muscles 6/7, since increased activity can drive synapse growth at the larval NMJ [37]. However, at present we do not have evidence to support this model. At the NMJ, however, all detectable Sdc is provided by the postsynaptic cell, and the molecular mechanisms of Sdc function at the synapse are ideally explored with post-synaptic expression of Sdc transgenes.

The overexpression and rescue experiments conducted in this study were done at 20°C in order to avoid the lethality observed when some of these constructs were expressed in a Sdc mutant background at 25°C. We hypothesize that this lethality may be due to fact that the Sdc constructs were cloned into the Attp2 site, which is the strongest expressor site in muscle [38]. We should note that the robust muscle expression observed for the Attp2 site might also be responsible for the more complete postsynaptic rescue when using Sdc-FL compared to the Sdc-wt construct used in previous studies. Combined with the observation that endogenously expressed Sdc is an HSPG [20] but overexpression can trigger the addition of CS sidechains [13] these data show that high levels of overexpression of HSPGs can change core protein glycosylation. Thus, in an attempt to avoid both the lethality of Sdc overexpression in muscles, and the possibility of inappropriate glycosylation on Sdc, we used reduced temperatures to decrease the activity of the Gal4-UAS system [39].

The requirement for *Drosophila* Sdc’s extracellular domain in Sdc function at the NMJ is likely due to its interaction with the receptor tyrosine phosphatase LAR [5]. The observation that overexpression of the ΔCyto construct causes a small but significant decrease in synapse size might be the result of a dominant negative function of the extracellular domains of Sdc. Such an effect is not without precedent, as previous studies in vertebrates have shown such effects [40, 41]. It is possible that the overexpression of the ΔCyto construct in muscle binds to presynaptic LAR, disrupting the interactions between LAR and the endogenously expressed Sdc that promote synapse growth.

This is the first study, however, to show a role for the cytoplasmic domains of *Drosophila* Sdc in Sdc function. Previous studies have shown that Sdc constructs lacking the highly conserved cytoplasmic domains are able to completely rescue Sdc function both at the CNS midline [13] and during tracheal cell migration [14]. Clearly, the functional requirement for *Drosophila* Sdc’s cytoplasmic domains does not appear to be a general phenomenon, but appears rather to be limited to Sdc function at the NMJ. Interestingly, in vertebrate systems, the cytoplasmic domains of Sdc are required for Sdc function both in terms of regulating actin cytoskeletal architecture [42, 43] and in promoting dendritic spine formation at the developing synapse [7].

Studies in vertebrates have made significant strides in understanding the molecular mechanisms of Sdc function at the developing synapse, with a particular emphasis on roles for the cytoplasmic domain. The C2 domain of vertebrate Sdc homologues interact with the PDZ domain of CASK [44] and can be coimmunoprecipitated in a complex with CASK from brain extracts [45]. Likewise, vertebrate Sdcs promote synapse growth postsynaptically through interactions of the C2 domain with the cytoplasmic protein Synbindin [7, 8] and through phosphorylation by the receptor tyrosine kinase EphB2 [9]. In addition, emerging evidence suggests that Sdc-2 appears to signal through the Sdc-2 binding partner neurofibromin to regulate protein kinase A (PKA) phosphorylation of Ena/VASP to promote synapse formation [17]. Thus, it would appear that in both *Drosophila* and vertebrates, the cytoplasmic domains of Sdc are crucial regulators of Sdc function at the synapse, and that Sdc functions postsynaptically in both.

Two models for the function of the cytoplasmic domain are consistent with our results. First, the cytoplasmic domains of Sdc might be required for localizing Sdc to the NMJ, and second, the cytoplasmic domains might bind to signaling proteins that regulate synapse growth on the postsynaptic side. Unfortunately, the reason that constructs lacking the cytoplasmic domains fail to promote synapse growth cannot be conclusively determined from the data in this study. In the presence of triton (Fig 3) all constructs lacking the cytoplasmic domain have moderate defects in their subcellular localization. Whether having reduced levels at the synapse (ΔCyto) or accumulations in the cytoplasm (ΔC1 and ΔC2), these data are consistent with a role for the cytoplasmic domains in Sdc localization. In the absence of triton, however, all constructs localize well to the synapse, demonstrating that the cytoplasmic domain is not necessary for synaptic localization. In addition, a comparison of Figs 3B and 4B also suggests that a complete deletion of the cytoplasmic domains of Sdc renders the ΔCyto construct sensitive to detergent extraction. Nevertheless, we cannot rule out the possibility that these constructs fail to promote synapse growth due to more subtle changes in their subcellular distributions. Examining the localization and function of candidate Sdc binding partners identified in this study might help to discriminate between these possibilities.

With the high degree of sequence conservation between the cytoplasmic domains of *Drosophila* and vertebrate Sdc, we anticipated obtaining some of the same binding partners for *Drosophila* Sdc that were found in vertebrates. Many of the binding partners in vertebrates bind to the C2 domain, which is 100% conserved between species [20] suggesting that likely binding partners would at least include the *Drosophila* homologues of CASK, Synbindin, Syntenin and Synectin. Yet, our screen did not yield *Drosophila* homologues of any of the vertebrate Sdc binding partners for either C1 or C2. The *Drosophila* genome encodes proteins with high sequence similarity to human synectin (*Drosophila* gene Kermit/CG11546–47% amino acid identity), synbindin (*Drosophila* gene CG9298–58% amino acid identity) and CASK/LIN2 (*Drosophila* gene Caki/CG6703–59% amino acid identity), but does not encode a protein with high similarity to syntenin. For CASK, this may be due to the fact that in vertebrates, interactions with Sdc’s cytoplasmic domains are dependent on Sdc dimerization [46] and our yeast two-hybrid bait protein was not dimerized.

While we recognize that our analysis of binding partners is based on an incomplete characterization of the clones obtained in our yeast two-hybrid screen, and that the vertebrate homologues may indeed be part of the pool of candidate binding partners, a more definitive assessment of whether the *Drosophila* homologues of CASK, synbindin and synectin bind to the cytoplasmic domains of *Drosophila* Sdc might better be made using candidate protein approach rather a yeast two-hybrid screen.

## Supporting Information

S1 Table Primers used for the generation of the Sdc constructs used in this study. (DOCX)

S2 Table Unabridged results from yeast two-hybrid screen. The 80 sequenced plasmids identified in the yeast two-hybrid screen are shown in alphabetical order. Genes that were obtained multiple times in the screen are shown multiple times in the table.
(DOCX)
