# Supplementary material for: The Extracellular and Cytoplasmic Domains of Syndecan Cooperate Postsynaptically to Promote Synapse Growth at the Drosophila Neuromuscular Junction
Source: PLoS One. 2016 Mar 17;11(3):e0151621. doi: 10.1371/journal.pone.0151621 (PMC4795781; doi:10.1371/journal.pone.0151621)
Supplement: S1 Table — (DOCX) [file pone.0151621.s001.docx]

| Primer Name | Primer Sequence |
| --- | --- |
| NotI 5' Sdc | AAAGAAGGAAATGCGGCCGCAACGGGGAAAATGAAGCCA |
| KpnI 3' Sdc | AAATGGTACCTCAGGCGTAGAACTCGCGG |
| KpnI 3'dltCytoSdc | AAATGGTACCTCACCTCATGCGGTACACG |
| KpnI 3' dltC2 Sdc | AAATGGTACCTCAGCGGTTATTCGCATTT |
| XhoI 3' SdcDT C1 | AATATACTCGAGGTACACGATGAACATGA |
| XhoI 5' SdcDT C1 | AAATAACTCGAGGCGCTGGACGAGCCAAA |
| XhoI5' Tm&C SdtEC | AATTATACTCGAGGACGATCGCACGTCGA |
| NotI5’ SMK SdtEC | AATTTATTGCGGCCGCTTTTGCAGGATCC |
| XhoI3' SMK SdtEC | AAATTATACTCGAGGGTGAGGTCGCCCAA |
| 3'PstIPdGFRXhoI: | TCGAGCCAAAGCATGATGAGGATGATAAGGGAGATGATGGTGAGCACCACCAGGGCCAGGATGGCTGAGATCACCACCACCTGCA |
| 5'PDGFRoligomer | GGTGGTGGTGATCTCAGCCATCCTGGCCCTGGTGGTGCTCACCATCATCTCCCTTATCATCCTCATCATGCTTTGGC |

**S1 Table. Primers used for the generation of the Sdc constructs used in this study.**
