# Supplementary material for: The Extracellular and Cytoplasmic Domains of Syndecan Cooperate Postsynaptically to Promote Synapse Growth at the Drosophila Neuromuscular Junction
Source: PLoS One. 2016 Mar 17;11(3):e0151621. doi: 10.1371/journal.pone.0151621 (PMC4795781; doi:10.1371/journal.pone.0151621)
Supplement: S2 Table — The 80 sequenced plasmids identified in the yeast two-hybrid screen are shown in alphabetical order. Genes that were obtained multiple times in the screen are shown multiple times in the table. (DOCX) [file pone.0151621.s002.docx]

**S2 Table. Unabridged results from yeast two-hybrid screen.** The 80 sequenced plasmids identified in the yeast two-hybrid screen are shown in alphabetical order. Genes that were obtained multiple times in the screen are shown multiple times in the table.

| Alan shepard | CG11963 | CG5205 | CG9083 | Cheerio | Ef2B | Helicase at 25E | Sec13 |
| --- | --- | --- | --- | --- | --- | --- | --- |
| Amalgam | CG13344 | CG5828 | CG9083 | Cheerio | Ef2B | Nelf-E | Sec13 |
| Bangles and Beads | CG15094 | CG5931 | CG9083 | Cheerio | Eif4a | Papilin | Shi |
| Bcop | CG18445 | CG6201 | CG9212 | Chord | Eif4a | Papilin | Taf10b |
| Brain tumor | CG30084 | CG6782 | CG9238 | Coracle | Eif4a | Papilin | TepIV |
| Ced-12 | CG3075 | CG7173 | CG9373 | Coracle | Eif4a | Papilin | Tom |
| Ced-12 | CG3328 | CG7173 | CG9413 | Cyclophilin | GCIM | Receptor of activated PKC | TROL |
| CG10005 | CG3989 | CG7927 | Cheerio | DAD | GRIP | Rpp20 | Tudor-SN |
| CG10005 | CG4656 | CG8176 | Cheerio | DAD | GRIP | Sallimus | Twin of M4 |
| CG10283 | CG4713 | CG8888 | Cheerio | EF2B | GRIP | Sallimus | Twin of M4 |
